# The neurobiology of survivorship: Cognitive consequences of modern oncology

**DOI:** 10.1093/noajnl/vdag121

**Published:** 2026-06-22

**Authors:** Maciej M Mrugala, Michelle Monje

**Affiliations:** Department of Neurology and Division of Medical Oncology, Mayo Clinic Comprehensive Cancer Center, Mayo Clinic, Phoenix, Arizona, USA; Department of Neurology and Neurological Sciences, Howard Hughes Medical Institute, Stanford University, Stanford, California, USA

**Keywords:** cancer treatment–related cognitive impairment (CRCI), chemotherapy, cognitive impairment, immunotherapy, radiotherapy

We are delighted to present to you the Special Issue of Neuro-Oncology Advances dedicated to treatment-related cognitive issues in oncology patients. While advances in cancer therapies have improved survival rates, oftentimes quite significantly, many survivors suffer from persistent cognitive side effects, frequently called “chemo-brain” or more appropriately, cancer treatment–related cognitive impairment (CRCI). Our understanding of this condition has significantly improved over the last several years with new mechanistic and therapeutic discoveries and will hopefully lead to new prevention and treatment strategies in the near future. In this special issue of Neuro-Oncology Advances, we gathered 4 articles from the pioneers in the field who share with us their research on CRCI.

Neth et al.^1^ describe the long-term consequences of cancer therapies in an authoritative review by pioneers in the field. CRCI is a cognitive syndrome that encompasses deficits in attention, executive function, memory, and processing speed, often persisting months or even years after treatment. While cognitive complaints have historically been linked to cranial radiation and cytotoxic chemotherapy, recent evidence also implicates immunotherapy in similar neurocognitive changes. These long-term cognitive impairments affect up to a third of all cancer survivors and can profoundly impair quality of life, occupational function, and psychosocial well-being.

The etiology of CRCI is multifactorial and likely involves both direct and indirect mechanisms. In chemotherapy, neurotoxicity arises from dysregulated neural-immune interactions, including microglial reactivity, oxidative stress, mitochondrial dysfunction, and consequent disruption of white matter oligodendroglial homeostasis and plasticity and hippocampal neurogenesis. Therapy-induced inflammation is increasingly recognized as a central player: microglial reactivity directly impairs hippocampal neurogenesis through cytokine signaling and results in disruption of oligodendroglial homeostasis and myelin plasticity. Underscoring the mechanistic role of neuroinflammation, microglial therapies or chemokine receptor blockade can rescue these cellular deficits and restore cognitive function in mouse models, and elevated cytokine/chemokine levels correlate with cognitive symptoms in patients. Neuroimaging studies support these biological underpinnings, showing alterations in white matter integrity, functional connectivity, and regional brain volumes such as the hippocampus.

In the elegant article juxtaposing chemotherapy-related neurotoxicity and aging, Nunez and Gibson^2^ walk us through the shared CNS pathologies underlying these two states and explore potential interventions. They propose that CRCI can be a model of induced aging and neurodegeneration, looking at shared CNS pathologies like neuroinflammation, reduced neurogenesis, disrupted myelination, and cellular senescence, to name a few. The parallel between aging and CRCI is a provocative and fascinating one. It allows us to draw from aging research and potentially develop targeted interventions for CRCI symptoms. The authors of this review identify that normal and chemotherapy-induced brain aging are accompanied by distinctive cellular and molecular markers. For example, it was discovered that breast cancer survivors who received chemotherapy are biologically older than age-matched non-cancer controls. The signs of aging can be seen by assessing the white matter volume, cortical thickness, gyrification, and sulcal depth in many areas of the brain.

By bridging the fields of neuro-oncology and gero-science, this review reframes CRCI not as an isolated toxicity but as a biological model of induced brain aging. The authors make a compelling case that interventions developed to counteract age-related neurodegeneration—such as anti-inflammatory therapies, senolytics, metabolic enhancers, and neuroprotective stimulation paradigms—could be repurposed or adapted to prevent or reverse CRCI.

Over the past several decades, the survival of children diagnosed with acute lymphoblastic leukemia (ALL) and primary brain tumors has improved dramatically.^3^^,^^4^ This remarkable progress, driven by therapeutic innovation and multidisciplinary care, has transformed what were once uniformly fatal diseases into conditions with growing populations of long-term survivors. Yet, as survival increases, attention has naturally shifted toward understanding and mitigating the chronic and often very debilitating late side effects of therapy. Among these, CRCI—particularly executive dysfunction—has emerged as one of the most pressing survivorship challenges.^4^^,^^5^

In this issue of Neuro-Oncology Advances, Alonso and Mabbott provide an outstanding review titled “Executive Function After Childhood Cancer: Insights from Network Neuroscience.”^6^ Their work synthesizes behavioral, neuroimaging, and neurobiological evidence linking the long-term cognitive effects of cancer and its treatments to alterations in the organization and function of large-scale brain networks. This review stands out not only for its depth and clarity but also for its integrative, systems-level perspective—an approach that will undoubtedly shape future research and clinical interventions in pediatric neuro-oncology. The authors seek to bridge behavioral and neural perspectives to offer insights that can inform clinical practices and improve outcomes for childhood cancer survivors.

Immunotherapy introduces novel immunological dimensions. CAR T cell immunotherapy results in neural-immune interactions that result in microglial reactivity, markedly elevated CNS cytokine/chemokine levels, and consequent disruption of hippocampal neurogenesis and oligodendroglial integrity. Similarly, immune checkpoint immunotherapies (ICIs), while unleashing cytotoxic T cells against tumors, can provoke neuroinflammatory cascades that can disrupt the blood-brain barrier and even induce paraneoplastic-like autoimmune encephalopathies. These effects underscore a growing need to characterize the neuroimmune interface in cancer treatment contexts.

A timely review at the cutting-edge of new cancer treatments by Hauswirth et al.^7^ discusses both acute and long-term effects of cellular immunotherapy (CAR T-cell and T cell engager therapies) on cognitive functions. Immune effector cell acute neurotoxicity syndrome (ICANS)—a transient syndrome that chiefly affects executive function, attention, and language—occurs frequently in patients treated with anti-CD9 CAR T cell therapy for hematological malignancies and is well-recognized. Long-term immunotherapy-related cognitive dysfunction (IRCI) is less well-described but increasingly recognized and shares both clinical features and mechanistic underpinnings^8^ with cancer therapy-related cognitive impairment (CRCI) following chemotherapy and radiotherapy.

As reviewed in this issue, advances in mechanistic, translational, and clinical CRCI research now position the field to advance therapeutic interventions for this debilitating consequence of cancer therapies and to improve quality of life for cancer survivors.

## Data Availability

The data underlying this article are available in the articles included in this special issue of Neuro-Oncology Advances and in its online version as well as in the references provided.
